# Maximal androgen blockade for the treatment of metastatic prostate cancer—a systematic review

**DOI:** 10.3747/co.v13i3.85

**Published:** 2006-06

**Authors:** H. Lukka, T. Waldron, L. Klotz, E. Winquist, J. Trachtenberg, on behalf of the Genitourinary Cancer Disease Site Group, of the Cancer Care Ontario Program in Evidence-based Care

**Affiliations:** * Juravinski Cancer Centre, 699 Concession Street, Hamilton, Ontario L8V 5C2; † Cancer Care Ontario Program in Evidence-based Care, McMaster University, 1280 Main Street West, Hamilton, Ontario L8S 4L8; ‡ Toronto–Sunnybrook Health Sciences Centre, 408–2075 Bayview Ave, Toronto, Ontario M4N 3M5; § London Health Sciences Centre, 790 Commissioners Road East, London, Ontario N6A 4L6; || The Princess Margaret Hospital, Department of Surgical Oncology, Division of Genito-Urinary Oncology, 610 University Avenue, Toronto, Ontario M5G 2C5; # Jack Barkin, Glenn Bauman, Julie Bowen, Michael Brundage, Joseph Chin, Richard Choo, Juanita Crook, Libni Eapen, Neil Fleshner, Barbara Markman, William Orovan, Kathleen Pritchard, Thomas Short, and John Srigley also contributed to the development of this systematic review through input in the discussion of the evidence. Please see the Cancer Care Ontario Program in Evidence-based Care Practice Guidelines Initiative Web site (www.cancercare.on.ca/access_PEBC) for a complete list of current Genitourinary Cancer Disease Site Members

**Keywords:** Prostatic neoplasms, androgen antagonists, hormonal anti-neoplastic agents

## Abstract

**Introduction:**

Maximal androgen blockade (mab) versus castration alone in patients with metastatic prostate cancer has been extensively evaluated in randomized trials. The inconsistent results have led to the publication of multiple meta-analyses. The present review examines the evidence from meta-analytic reports to determine whether mab using agents such as flutamide, nilutamide, and cyproterone acetate (cpa) is associated with a survival advantage.

**Methods:**

We conducted a systematic review of the literature (medline, embase, and the Cochrane Library through July 2004; cancerlit through October 2002) for meta-analyses that compared mab with castration alone in previously untreated men with metastatic prostate cancer (D1 or D2, N+/M0 or M1). Two reviewers selected papers for eligibility; disagreement was resolved by all the authors through consensus.

**Results:**

The literature search identified six meta-analyses that met the eligibility criteria of the review. Two of those reports were based on individual patient data (ipd), and four were based on data from the published literature. All six meta-analyses pooled data on overall survival.

The best evidence came from the largest meta-analysis, conducted by the Prostate Cancer Trialists Collaborative Group and based on ipd (8725 patients) from 27 trials. That analysis detected no difference in overall survival between mab and castration alone at 2 or 5 years. However, a subgroup analysis showed that mab with nonsteroidal anti-androgens (nsaas) was associated with a statistically significant improvement in 5-year survival over castration alone (27.6% vs. 24.7%; *p* = 0.005). The combination of mab with cpa, a steroidal anti-androgen, was associated with a statistically significant increased risk of death (15.4% vs. 18.1%; *p* = 0.04). Compared with castration alone, mab was associated with more side effects (that is, gastrointestinal, endocrine function) and reduced quality of life in domains related to treatment symptoms and emotional functioning.

**Conclusions:**

The small survival benefit conferred by mab with nsaa is of questionable clinical significance given the added toxicity and concomitant decline in quality of life observed in patients treated with mab. Therefore, combined treatment with flutamide or nilutamide should not be routinely offered to patients with meta-static prostate cancer beyond the purpose of blocking testosterone flare. Monotherapy, consisting of orchiectomy or the administration of a luteinizing hormone–releasing hormone agonist is recommended as standard treatment.

## 1. INTRODUCTION

Prostate cancer is currently the most prevalent form of male cancer in Canada 1. At diagnosis, 20%–30% of patients will present with advanced or metastatic disease. Of those men, approximately 25% will die from their disease within 2 years 2. Therapeutic interventions seek not only to increase survival in those patients, but also to improve quality of life (qol) 3.

The mainstay of treatment for advanced or meta-static prostate cancer is to inhibit the biosynthesis of androgens, the hormones responsible for prostate cancer cell growth. Androgen suppression can be achieved through surgical (bilateral orchiectomy) or medical castration. Medical castration involves the long-term use of luteinizing hormone–releasing hormone (lhrh) agonists. The two methods of castration appear equally effective in removing testicular androgens 4.

The testes are the major locale for testosterone production; however, the adrenal glands also produce a small but measurable quantity of androgens. It has been hypothesized that removing all circulating androgens—by blocking adrenal androgens in addition to inhibiting testicular androgen production—might be beneficial to patients. Combination treatment, in the form of surgical or medical castration plus administration of an anti-androgen [for example, flutamide, nilutamide, or cyproterone acetate (cpa)] is called “maximal androgen blockade” (mab).

The use of mab was first introduced in the early 1980s 5. Since then, a large number of randomized controlled trials have been conducted to evaluate the efficacy of mab as compared with castration alone. The trials yielded inconsistent results. Most failed to provide convincing evidence of improved survival with mab; however, a few of the larger trials detected survival benefits with combined treatment 6–8. Low statistical power, study immaturity, compliance to treatment, and imbalances in prognostic indicators between study arms of individual trials were implicated as potential sources of discrepancy 9–13.

Recent attempts to determine the treatment efficacy of mab have involved meta-analyses of the trials 14. To determine whether mab is associated with a survival advantage, the present review systematically examines the results of the meta-analyses comparing mab with castration alone in patients with metastatic prostate cancer.

## 2. MATERIALS AND METHODS

The present systematic review was originally completed in the context of developing a clinical practice guideline for Cancer Care Ontario’s Program in Evidence-Based Care (pebc), using the methodology of the Practice Guidelines Development Cycle 15. The literature was searched by one member of pebc’s Genitourinary Cancer Disease Site Group. Evidence was reviewed and selected by two members, and disagreements pertaining to eligibility were handled through consensus involving the five members of the writing group. Two reviewers assessed eligible reports for important aspects of methodologic quality as expressed in the Quorom statement 16 (Appendix A).

### 2.1 Literature Search Strategy

We conducted a systematic search of medline (1980 through July 2004), embase (1980 through 2004 wk 27), cancerlit (1980 through October 2002), and the Cochrane Library (2004, Issue 2) databases. In each database, subject headings were combined with disease-specific, treatment-specific, and design-specific search terms (Appendix B). The reference lists of all articles found, including reviews and articles held in personal files, were reviewed for additional citations. The search was restricted to reports published in the English language.

### 2.2 Eligibility Criteria

Published reports or abstracts of meta-analyses comparing mab (orchiectomy or lhrh agonist plus administration of an anti-androgen) with castration alone (orchiectomy or lhrh agonist) in previously untreated men with metastatic prostate cancer (D1 or D2, N+/M0 or M1) were eligible for inclusion. Papers were required to report overall mortality or disease progression-related outcomes, or both. Adverse effects and qol were also outcomes of interest.

## 3. RESULTS

### 3.1 Literature Search Results

We identified eleven reports representing seven unique meta-analyses 17–27. One meta-analysis was excluded based on language 27, leaving six analyses eligible for inclusion in the review 18,20,22–24,26 (Table I). Two meta-analyses pooled individual patient data (ipd) 18,26, and four pooled summary data from published trial reports (literature-based) 20,22–24.

#### 3.1.2 IPD Meta-analyses

Bertagna *et al.* 25 published the first ipd meta-analysis in 1994. That analysis was limited to seven double-blind, placebo-controlled trials of mab with nilutamide (1056 patients). An update published in abstract form by Debruyne *et al.* 26 provided extended follow-up data on survival and disease progression.

In 1995, the Prostate Cancer Trialists’ Collaborative Group (pctcg) published an ipd meta-analysis that included 22 mab trials (5710 patients) 17. All randomized trials that compared castration alone to mab, both published and unpublished, were sought for inclusion. The main limitations of the report include the absence of explicitly defined eligibility criteria, a description of the methods used to identify and select trials, an appraisal of trial quality and its influence on the pooled results, and an indication of whether subgroup analyses were planned *a priori* (Appendix A). The report is also limited by the fact that overall mortality was the only outcome analyzed; other important endpoints, including toxicity and qol were not examined. The meta-analysis was updated in 2000 18 to include a total of twenty-seven trials: twelve used flutamide, eight used nilutamide, and seven used cpa as the anti-androgen. In combining data on 8725 patients, this updated report represents the most extensive quantitative analysis of mab trials conducted to date.

#### 3.1.3 Literature-based Meta-analyses

The number of trials included in the four literature-based meta-analyses ranged from nine (1978 patients) to twenty (6745 patients; Table I). The largest analysis was conducted for the Agency for Health Care Policy Research (ahcpr) by Aronson *et al.* 20. The review was well conducted, with trials systematically identified through a prospectively designed protocol that specified the objectives, literature search strategy, eligibility criteria, method of assessing trial quality, and subgroup and sensitivity analyses, including an assessment of publication bias (Appendix A). Twenty-seven mab trials were identified, including twelve using flutamide, eight using nilutamide, and seven using cpa. Overall mortality was the only outcome for which data were statistically pooled, but data on disease progression, qol, and adverse effects were also summarized.

The three other literature-based meta-analyses were restricted in scope, analyzing trials that compared castration with mab using nonsteroidal anti-androgens (nsaa) 22–24. The largest of those analyses, carried out by Schmitt *et al.* 22 for the Cochrane Prostatic Diseases and Urologic Cancers Group, included twenty trials and pooled data on overall mortality, progression-free survival (pfs), and cancer-specific survival. Bennett *et al.* 23 and Caubet *et al.* 24 both included nine trials and pooled data on overall mortality 23,24 and pfs 24.

### 3.2 Outcomes

#### 3.2.1 Overall Survival

##### IPD Meta-analyses

Results from the original pctcg overview showed a small survival benefit with mab that was not statistically significant (5-year survival: 22.8% vs. 26.2%; *p* > 0.1) 17. In the updated meta-analysis (2000), the pctcg reported a nonsignificant overall hazard ratio (hr) of 0.96 [95% confidence interval (ci): 0.91–1.01; *p* = 0.11], where ratios less than 1 favoured mab 18 (Table II). Further analyses at different follow-up periods also showed no difference in mortality and suggested an absolute 5-year survival difference of approximately 2% in favour of mab. Subgroup analyses were performed by method of androgen suppression (orchiectomy vs. lhrh agonist), type of anti-androgen, patient age, stage of disease (metastases vs. no metastases), and non–prostate cancer mortality. With the exception of type of anti-androgen, no significant differences in treatment effect were observed within any of those subgroups. A small and statistically significant survival benefit was detected for mab with flutamide (hr = 0.92; 95% ci: 0.86–0.98; *p* = 0.02), and a similar but nonsignificant result was observed for nilutamide. mab with cpa was associated with a significantly worse survival outcome than castration alone (hr = 1.13; 95% ci: 1.01–1.25; *p* = 0.04). Treatment with mab containing either of the nsaas increased 5-year survival over castration alone by 3% (27.6% vs. 24.7%, *p* = 0.005).

Debruyne *et al.* 26 reported a reduction in the odds of death in patients treated with nilutamide-containing mab; mab was associated with a 16% reduction in mortality as compared with castration alone [odds ratio (or) = 0.84; 95% ci: 0.71–0.99; *p* = 0.038].

##### Literature-based Meta-analyses

Table III summarizes the results for overall mortality from the four literature-based meta-analyses. Aronson *et al.* 20 detected no significant difference in overall mortality at 2 years, although at 5 years, overall mortality was significantly improved with mab (hr = 0.87; 95% ci: 0.81–0.94). However, the 5-year estimate was based on half the trials (10 trials, 66% of patients) that contributed to the 2-year estimate. No differences in treatment effect were detected in any of the subgroup analyses performed (method of androgen suppression, stage of disease, type of anti-androgen, or trial quality).

Schmitt *et al.* 22 reported no difference in mortality at 1 or 2 years between nsaa mab and castration-only arms, but 5-year mortality was better with mab (or = 1.29; 95% ci: 1.11–1.50; *p* = 0.0009). The two other literature-based reports examining nsaa mab detected significant reductions in the risk for mortality with mab that ranged between 10% and 22% 23,24.

#### 3.2.2 Disease Progression

Pooled analyses of disease progression data were available from three of the six meta-analyses 22,24,26; however, those analyses are limited by the inclusion of a small proportion of mab trials. Each of those reports combined data from trials of mab using nsaa. Debruyne *et al.* 26 reported that, among seven trials, the odds of progression were reduced by 17% by mab with nilutamide (or = 0.83; 95% ci: 0.70–0.98; *p* = 0.031). Schmitt *et al.* 22pooled published pfs data at 1 (seven trials), 2 (five trials), and 5 years (two trials); the odds of progression were significantly reduced with mab at 1 year (or = 1.38; 95% ci: 1.15–1.67; *p* = 0.0006), but not at 2 or 5 years. Caubet *et al.* 24 reported a 23%–26% reduction in the risk for progression with mab depending on the type of meta-analytic method used [relative risk (rr) = 0.74, *p* < 0.001 among seven trials; rr = 0.77, *p* < 0.001 among seven trials].

A more representative presentation of disease progression data was provided by Aronson *et al.* 20. They summarized twenty-three trials 6,8,29,30,32,36,38–40,42,43,46–49,53–58,60 reporting those data. Nineteen of the trials reported no significant difference between mab and castration alone on those measures 29,30,32,36,38–40,42,43,46–49,54–58,60. Among six trials reporting pfs 6,8,29,32,36,47, two reported a statistically significant longer progression-free interval with mab 6,8. Both of those trials used an nsaa in the mab arm. One trial compared orchiectomy plus placebo with orchiectomy plus nilutamide (median pfs: 15 months vs. 21 months; *p* = 0.002) 8, and the other compared leuprolide plus placebo with leuprolide plus flutamide (median pfs: 14 months vs. 17 months; *p* = 0.039) 6.Of the seventeen trials that reported on time-to-disease progression (ttp) 4,30,38–40,42,43,46,48,49, 54–56,58,60, one trial comparing orchiectomy with goserelin plus flutamide detected a significantly longer ttp interval with mab (median ttp: 18 months vs. 12 months; *p* = 0.002) 4.

#### 3.2.3 QOL and Adverse Effects

Aronson *et al.* 20 wrote the only report that reviewed qol and adverse effects. Among the 27 randomized controlled trials that those authors reviewed, only one formally assessed qol. The authors summarized two reports 61,62 of the large National Cancer Institute (nci) int-0105 Southwest Oncology Group/Eastern Cooperative Oncology Group trial 29, which compared orchiectomy plus flutamide with orchiectomy plus placebo. Measures of qol included three treatment-related symptoms (diarrhea, gas pain, and body image), physical functioning, and emotional functioning; all were assessed at 1, 3, and 6 months after the start of treatment. Patients treated with mab experienced significantly more diarrhea at 3 months (*p* < 0.001) and worse emotional functioning at 3 and 6 months (*p* < 0.003) than did patients treated with castration alone. Nonsignificant trends toward poor scores on measures of physical functioning, fatigue, abdominal gas, overall pain, and body image were also observed with mab.

Major limitations were present in the reporting of adverse effects in the mab trials. Because of those limitations, data on adverse effects were supplemented with similar data from package inserts that accompany therapeutic agents marketed in the United States and from phase ii studies. Tables IV and V summarize the adverse effects data contained in the Aronson *et al.* report 20. Compared with castration alone, treatment with mab that included nsaa appeared to produce more gastrointestinal-related problems and more frequent withdrawal from treatment. When mab contained cpa, treatment was associated with more complications related to endocrine function, but a withdrawal pattern similar to that in patients receiving monotherapy was demonstrated.

## 4. DISCUSSION

Six meta-analyses form the evidence base of the present review 17,18,20,22–25. Evidence from those analyses suggests that patient outcomes depend on the type of anti-androgen used with mab. The pctcg meta-analysis 18 showed that mab was not associated with a statistically significant improvement in overall survival. However, when outcomes were analyzed by type of anti-androgen, varying treatment efficacies among the agents were evident. Small but statistically significant survival benefits in the range of 3% at 5 years were detected among trials that used mab with an nsaa (as compared with castration alone). Compared with castration alone, mab with cpa (a steroidal anti-androgen) was associated with an approximate 3% increased risk of death.

Variability in the magnitude of outcome among meta-analyses may arise from a number of factors, including the number and size of the trials contributing to the pooled estimate, the type of anti-androgens being evaluated, and the use of published summary data or ipd for the analyses. The four literature-based meta-analyses 20,22–24 included fewer trials (and fewer patients) than did the pctcg meta-analysis, but the resulting pooled estimates were of greater magnitude (in favour of mab) than were those generated using ipd 18. In meta-analyses based on published data, publication bias is more likely to exaggerate treatment effects 63. Only one of the four literature-based meta-analyses assessed the influence of publication status on the overall pooled result 20. With ipd, many of the problems associated with published data that introduce bias are eliminated by the ability to incorporate all trial data (published and unpublished), to check the integrity of patient randomization, and to perform proper time-to-event analyses (as compared with fixed time point) by intent-to-treat 64,65. Further, because of greater patient numbers, ipd often provides greater statistical power to properly perform subgroup analyses 65. The methodologic weaknesses of the pctcg have been identified, but the advantages of using ipd currently make the pctcg meta-analysis the most reliable evidence comparing mab with castration alone.

To decide whether mab should be the preferred treatment for patients, the small survival benefit and the additional adverse effects of combined treatment must be balanced. The clinical significance of a statistically significant 3% improvement in survival with nsaa mab is questionable, especially when the toxicity of mab is considered. Data on adverse effects and qol are limited, but they suggest increased toxicity and a concomitant decline in qol in mab-treated patients. In addition, data on disease progression provide further evidence that mab does not provide superior treatment efficacy over castration alone 20.

Based on the evidence reviewed, mab should not be routinely offered to patients with metastatic prostate cancer. Monotherapy, consisting of orchiectomy or the administration of an lhrh agonist, should be recommended as standard treatment.

It is important to distinguish between mab as long-term treatment and short-term use of mab in the prevention of testosterone flare. In patients treated with medical castration, initial treatment with an lhrh agonist is accompanied by a surge in serum testosterone during the first week or weeks of therapy, followed by a decline. That surge may exacerbate existing metastatic disease 66,67,68, therefore short-term use of an anti-androgen is indicated to prevent or block the flare phenomenon 68. Administration of an anti-androgen is reasonable for a period of 2–4 weeks when treatment with an lhrh agonist is initiated.

Because of the small survival improvement observed with mab, some clinicians may still choose mab over monotherapy for individual patients. If mab is administered with this intent, mab containing a nsaa is suggested. Given its higher mortality, mab with cpa should be avoided as compared with castration alone 18.

The present review did not identify any meta-analyses that included trials evaluating mab with the newer anti-androgen bicalutamide. Bicalutamide-based mab has been compared in a randomized trial only with combination flutamide 69. A castration-only control arm was deemed unethical at the time the bicalutamide trial was designed because mab was considered standard care (over monotherapy). The trial compared bicalutamide plus an lhrh agonist with flutamide plus an lhrh agonist and detected a survival improvement with bicalutamide that was not statistically significant (median survival: 180 weeks vs. 148 weeks; hr = 0.87; 95% ci: 0.72–1.05; *p* = 0.15) 69. With the exception of a higher incidence of hematuria, bicalutamide appeared less toxic than flutamide. Klotz *et al.* 70 recently re-analyzed the data from the bicalutamide trial 69 and the pctcg meta-analysis (subgroup of trials comparing mab with flutamide versus castration alone) 18 to calculate an estimate of the likely benefit of mab with bicalutamide relative to castration alone. They reported an estimated hr of 0.80 (95% ci: 0.66–0.98) for bicalutamide-based mab versus castration alone, which equates to a 20% relative reduction in the risk of death with bicalutamide. On the basis of those data, use of bicalutamide in patients who are offered mab would also be reasonable. A randomized trial comparing mab with bicalutamide to castration alone is ongoing, but that trial is assessing bicalutamide at a dose of 80 mg 71. Before beginning mab, selected patients should be advised of the magnitude of the survival benefit and on possible adverse effects and their potential impact on qol.

Progressive prostate cancer is usually detected through a rise in prostate-specific antigen (psa), which usually predates clinical or radiologic evidence of metastases. Most patients included in mab trials had documented metastases (stage D2), and whether results from those trials are generalizable to patients with a rising psa without evidence of metastatic disease is unknown. Only a handful of trials have analyzed outcomes by extent of meta-static involvement 4,6,11,29,34. Most of those have not shown a benefit of mab in patients with minimal disease, although the subgroup analyses included small numbers of patients. Only 12% of patients (approximately 1000) in the pctcg meta-analysis 18 had documented non-metastatic prostate cancer. An analysis of those patients showed slightly worse survival with mab, although the difference did not reach statistical significance. Prospective randomized trials to investigate the efficacy of mab in that subgroup of patients are warranted.

## 5. CONCLUSIONS

The small survival benefit conferred by mab with nsaa is of questionable clinical significance given the added toxicity and concomitant decline in qol observed in patients treated with mab. Therefore, combined treatment with flutamide or nilutamide should not be routinely offered to patients with metastatic prostate cancer (beyond the purpose of blocking testosterone flare). Monotherapy consisting of orchiectomy or the administration of a lhrh agonist is recommended as standard treatment.

## Figures and Tables

**Table I:** Meta-analyses identified by the literature search—descriptions

	*Trials included in meta-analysis:mabwith*		
*Meta-analysis*	*Flutamide*	*Nilutamide*	*cpa*
Individual patient data (ipd) meta-analyses			
pctcg 2000 18	Bono (ilg ), 1998^28^	Dijkman (iasg), 1997^8^	De Voogt (eortc), 1998^44^
31 rcts included in review	Denis (eortc), 1998 7	Bertagna, 1994^25^	Theiss, 1996^45^
27 rcts provided ipd for meta-analysis	Eisenberger (nci/swog), 1998 29	Béland (casg), 1990^38^	Thorpe, 1996^46^
12 flutamide trials	Ferrari (Italy), 1996^30^	Crawford, 1990^39^	Robinson (eortc), 1995^47^
8 nilutamide trials	Zalcberg (Australia), 1996 31	Namer, 1990^40^	Jorgensen (spcg ), 1993^48^
7 cpa trials	Boccardo (poncap), 1993^32^	Knonagel, 1989^41^	DiSilverio (Italy), 1990^49^
88% of patients staged “metastatic”;	Fourcade (France), 1993 33	Brisset, 1987 42	
12% staged “locally advanced”	Iversen (daproca), 1993 34 Tyrrell (ipcsg ), 1993 35 Crawford (nci), 1989^6^ Schulze (wpsg), 1988 36 Delaere, 1987 37	Navratil, 1987 43	
Bertagna 1994 25, Debruyne 1996 26	None	Bertagna, 1994 25	None
Included 7 double-blind rcts		Janknegt (iasg), 1993^50^	
7 nilutamide trials		Brisset, 1990 51	
% of patients staged D not reported		Namer, 1990 40 Knonagel, 1989 41 Béland (casg), 1988 52	
Literature-based meta-analyses			
Schmitt 2003 22	Bono (ilg), 1998^28^	Dijkman (iasg), 1997^8^	None
20 rcts included in review	Eisenberger (nci/swog/int- 1015), 1998 29	Béland (casg), 1990^38^	
14 rcts provided data for meta-analysis a		Crawford, 1990 39	
9 flutamide trials	Zalcberg (Australia), 1996 31	Namer (France), 1990 40	
5 nilutamide trials	Boccardo (poncap), 1993 32	Brisset, 1987 42	
96% of patients were stage D2 or M1	Denis (eortc), 1993^53^	Tyrrell (ipcsg), 1991^54^ Fourcade (France), 1990 55 Iversen (daproca), 1990 56 Crawford (nci), 1989 6	
Aronson 1999^20^	Same as above	Same as above	Robinson (eortc), 1995^47^
27 rcts included in review			Jorgensen (spcg), 1993 48
20 rcts provided data for meta-analysis			DeVoogt (eortc ), 1990^57^
9 flutamide trials			DiSilverio (Italy), 1990 49
5 nilutamide trials			Williams (U.K.), 1990 58
6 cpa trials			Klosterhalfen, 1987 59
93% of patients were stage D2			
Bennet 1999 23	Eisenberger (nci/swog), 1998^29^	None	None
9 rcts included in review	Zalcberg (Australia), 1996^31^		
9 rcts provided data for meta-analysis	Boccardo (poncap), 1993^32^		
9 flutamide trials	Denis (eortc), 1993 4		
98% of patients were stage D	Fourcade (France), 1993 33 Tyrrell (ipcsg), 1991 54 Iversen (daproca), 1990 56 Crawford (nci), 1989^6^ Schulze (wpsg), 1988 36		
Caubet 1997 24	Boccardo (poncap), 1993^32^	Janknegt (iasg), 1993^50^	None
13 rcts included in review	Denis (eortc), 1993 4	Béland (casg), 1991^38^	
9 rcts provided data for meta-analysis	Iversen (daproca), 1993 34	Navratil, 1987^43^	
6 flutamide trials	Tyrrell (ipcsg), 1993 35		
3 nilutamide trials	Crawford (nci), 1989^6^		
57%–100% of patients staged D2	Schulze (wpsg), 1988 36		

a The 14 trials listed in Table I contributed to the pooled analysis of 2-year survival data. Thirteen trials 6,8,28,29,31,32,34,38,40,42,53–55 and seven trials 6,8,28,29,34,53,54 contributed to the pooled analysis of 1-year and 5-year survival data, respectively.

mab = maximal androgen blockade; cpa = cyproterone acetate; pctcg = Prostate Cancer Trialists’ Collaborative Group; rcts = randomized controlled trials; ilg = Italian Leuprorelin Group; eortc = European Organization for Research and Treatment of Cancer; nci = National Cancer Institute; swog = Southwest Oncology Group; poncap = Italian Prostatic Cancer Project; daproca = Danish Prostatic Cancer Group; ipcsg = International Prostate Cancer Study Group; wpsg = Westfälische Prostatakarzinom Study Group; iasg = International Anandron Study Group; casg = Canadian Anandron Study Group; spcg = Scandinavian Prostatic Cancer Group.

**Table II:** Results (mortality for maximal androgen blockade vs. castration alone ^a^) from the 2000 Prostate Cancer Trialists’ Collaborative Group individual patient data meta-analysis

	*Estimatedhr(95%ci),* p *value*
Overall mortality (*n=*8725)	0.96 (0.91–1.01), 0.11
By years since randomization:	
Year 0	1.01 (0.91–1.11), nr
Years 1–2	0.93 (0.85–1.01), nr
Years 3–4	0.97 (0.85–1.09), nr
Year 5 onward	0.94 (0.76–1.12), nr 5-Year survival estimates: 25.4% vs. 23.6%, *p>*0.1
Subgroup analyses	
Age	
<65 (*n=*1641)	0.90 (0.78–1.02), nr
65–74 (*n=*3094)	0.96 (0.88–1.04), nr
≥75 (*n=*2487)	0.95 (0.85–1.05), nr
Stage of disease	
Definite metastases (*n=*7190)	0.95 (0.89–1.01), nr
No metastases (*n=*1025)	1.06 (0.86–1.30), nr
Non-prostate cancer mortality (*n=*4876)	1.04 (0.88–1.20), 0.7
Type of anti-androgen	
Flutamide (*n=*4803)	0.92 (0.86–0.98), 0.02
Nilutamide (*n=*1751)	0.92 (0.80–1.04), >0.1
cpa (*n=*1661)	1.13 (1.01–1.25), 0.04
Class of anti-androgen	5-Year survival estimates:
Non-steroidal (flutamide + nilutamide; *n=*6500)	27.6% vs. 24.7%, *p=*0.005
Steroidal (cpa; *n=*1800)	15.4% vs. 18.1%, *p=*0.04

hr = hazard ratio; ci = confidence interval; nr = not reported; cpa = cyproterone acetate.

**Table III:** Results from literature-based meta-analyses of maximal androgen blockade (mab)

*Meta-analysis*	*Mortality results [estimated*hr*(95%ci),* p *value]*		
mab with any anti-androgen vs. castration alone a			
Aronson 1999 20		2-year (20 trials, 6745 patients) 0.97 (0.87–1.09), nr	5-year (10 trials, 4443 patients) 0.87 (0.81–0.94), nr
mab with non-steroidal anti-androgens vs. castration alone a			
Schmitt 2003 22	1-year (13 trials, 4970 patients) 1.03 b (0.85–1.25), 0.7	2-year (14 trials, 5286 patients) 1.14 b (1.00–1.31), 0.06	5-year (7 trials, 3550 patients) 1.29 b (1.11–1.50), 0.0009
Bennet 1999^23^	Overall (9 trials, 4128 patients) 0.90 (0.79–1.00), nr		
Caubet 1997 24	Overall: Method 1 c (7 trials, 1978 patients) 0.78 (0.67–0.90), <0.001 Overall: Method 2 c (7 trials, 2592 patients) 0.84 (0.76–0.93), <0.001		

a Unless otherwise specified, summary statistic values less than 1 favour mab and values greater than 1 favour castration alone.

b Odds ratio. A ratio greater than 1 favours mab; a ratio less than 1 favours castration alone.

c Method 1: Hazard ratios were derived by reconstructing annual life tables from survival curves and fitting discrete proportional hazard models. Method 2: Hazard ratios were derived from reported *p* values and numbers of deaths.

hr = hazard ratio; ci = confidence interval; nr = not reported.

**Table IV:** Adverse effects by category, combined results

	*Castration only*		*mab: Castration +nsaa*		*mab: Castration +cpa*		*Anymab*	
*Adverse effect*	*Patients [*n *(%)]*	*Studies (*n*)*	*Patients [*n *(%)]*	*Studies (*n*)*	*Patients [*n *(%)]*	*Studies (*n*)*	*Patients [*n *(%)]*	*Studies (*n*)*
Cardiovascular								
Cardiovascular, not specified	570 (4)	4	387 (4.9)	3	175 (1.7)	1	562 (3.9)	4
Edema	569 (3.2)	3	293 (2)	1	277 (6.5)	2	570 (4.2)	3
Endocrine								
Hot flashes	2594 (40.1)	16	2789 (40)	12	488 (52.7)	4	3277 (41.9)	16
Gynecomastia	1441 (9.4)	10	1987 (7)	9	257 (17.5)	2	2244 (8.2)	11
Breast tenderness or pain	649 (7.7)	5	1206 (5.1)	5	257 (6.6)	2	1463 (5.4)	7
Impotence	515 (71.1)	5	362 (66)	4	156 (82.1)	1	518 (70.8)	5
Decreased libido	519 (70.1)	5	367 (65.4)	4	156 (78.8)	1	523 (69.4)	5
Gastrointestinal (gi)								
gi, not specified	959 (2.3)	7	768 (10.3)	6	175 (0.6)	1	943 (8.5)	7
Nausea or vomiting	1872 (3.2–7.1)	8	1851 (5.6–9.2)	8	0 (0)	0	1851 (5.6–9.2)	8
Diarrhea	1464 (2.2)	6	1458 (8.2)	6	0 (0)	0	1458 (8.2)	6
gi pain	124 (1.6)	2	122 (7.4)	2	0 (0)	0	122 (7.4)	2
Hepatic								
Hepatic, not specified	1197 (1.3)	4	2004 (5)	6	0 (0)	0		
Increased liver enzymes	483 (2.7)	3	474 (6.8)	3	0 (0)	0	nr	
*Ophthalmologic*	*Orchiectomy*		*Orchiectomy + nilutamide*					
Ophthalmologic, not specified	407 (5.4)	3	396 (29)	3	nr		nr	

mab = maximal androgen blockade; nsaa = nonsteroidal anti-androgen; cpa = cyproterone acetate; nr = not reported. Adapted, with permission, from Aronson *et al.*^20^, Appendix ii, Tables ii-6 to ii-10.

**Table V:** Adverse effects leading to withdrawal from treatment, combined results

*Treatment*	*Studies (*n*)*	*Treatment group (*n*)*	*Withdrawals from treatment [*n *(%)]*
Leuprolide (1 daily)	1	268	0 (0)
Goserelin (3.6, 1-month)	11	1679	33 (2)
Goserelin (10.8, 3-month)	2	77	1 (1.3)
Buserelin (0.4)	1	72	3 (4.2)
Orchiectomy + nilutamide (150)	2	271	38 (14)
Orchiectomy + nilutamide (300)	3	209	24 (11.5)
Orchiectomy + cpa (150)	1	102	3 (2.9)
Orchiectomy + cpa (300)	1	20	2 (10)
Orchiectomy or lhrh agonist or both	28	4275	82 (1.9)
Goserelin (3.6, 1-month) + flutamide (750)	5	846	94 (11.1)
Orchiectomy or lhrh agonist + flutamide (750) or both	9	2804	233 (8.3)
Orchiectomy or lhrh agonist + bicalutamide (50) or both	1	401	41 (10.2)
Orchiectomy or lhrh agonist + nilutamide (150 or 300) or both	5	480	62 (12.9)
Orchiectomy or lhrh agonist + cpa (150 or 300) or both	2	122	5 (4.1)

cpa = cyproterone acetate; lhrh = luteinizing hormone–releasing hormone. From Aronson *et al.* 20, Appendix ii, Table ii-11. Used with permission.

## Appendix A Meta-analysis quality, as evaluated using the quality of reporting of meta-analyses (Quorom) statement; closed circles denote fully described items, open circles denote partially described items, and dashes denote items not described

**Table:**

	*Meta-analyses*					
	*ipd*		*Literature-based*			
*Quorom checklist item*	*pctcg**2000**18**,**pctcg**1995**17*	*Bertagna 1994**25**, Debruyne 1996**26*	*Schmitt2003**22*	*Aronson 1999**20*	*Bennett 1999**23*	*Caubet 1997**24*
INTRODUCTION						
Clinical problem	—	—	•	•	•	—
Biologic rationale for treatment	•	•	•	•	•	•
Rationale for review	•	•	•	•	•	•
METHODS						
Searching						
Information sources (e.g., databases, registers)	○	—	•	•	—	•
Restrictions (e.g., years, publication status, language)	○	—	○	•	○	•
Selection						
Inclusion/exclusion criteria (e.g., defining population, intervention, outcomes, and study design)	○	○	•	•	○	•
Validity assessment						
Criteria and process used (e.g., masked conditions, quality assessment, and their findings)	—	—	•	•	—	•
Data abstraction						
Process used (e.g., completed independently or in duplicate)	—	—	•	•	○	•
Study characteristics						
Type of study design	•	•	•	•	—	—
Participants’ characteristics	—	•	•	•	—	—
Details of intervention	—	•	•	•	—	—
Outcome definitions	—	•	•	•	○	—
How clinical heterogeneity assessed	—	—	—	•	—	—
Quantitative data synthesis						
Measures of effect (e.g., relative risk, hazard ratio)	•	•	•	•	•	•
Method of combining results (e.g., statistical testing, cis)	•	•	•	•	•	•
Handling of missing data	—	—	—	•	•	•
How statistical heterogeneity was assessed	—	•	•	•	—	—
Rationale for any *a priori* sub-group and sensitivity analyses	—	—	•	•	—	•
Assessment of publication bias	—	—	—	•	—	—
RESULTS						
Trial flow						
Provide meta-analysis profile summarizing trial flow	—	—	—	—	—	—
Study characteristics						
Present descriptive data for each trial (e.g., age, sample size, intervention, dose, duration, follow-up period)	•	—	•	•	•	•
Quantitative data synthesis						
Report agreement on selection and validity assessment	—	—	•	•	—	•
Present summary results (for each treatment group in trial and each outcome)	•	•	•	•	•	—
Present data needed to calculate effect sizes and cis in itt analyses (e.g., 2×2 tables of counts, means, proportions, sds)	•	•	•	•	—	—
DISCUSSION						
Summarize key findings	•	•	•	•	•	•
Discuss clinical inferences based on internal and external validity	•	○	•	•	•	•
Interpret results in light of the totality of evidence	•	—	○	○	•	•
Describe potential biases in the review process (e.g., publication bias)	•	—	○	○	○	○
Suggest future research agenda	—	—	•	•	—	○

ipd = individual patient data; pctcg = Prostate Cancer Trialists’ Collaborative Group; cis = confidence intervals; itt = intent-to-treat; sds = standard deviations.

## Appendix B Literature search strategy

**Table:**

*medline*	*embase*
1. practice guidelines/	1. exp randomized controlled trial/
2. practice guideline.pt.	2. exp controlled study/
3. practice guideline?.ti,tw.	3. Major Clinical Study/
4. meta-analysis/	4. Clinical trial/
5. metaanal:.ti,tw.	5. or/1–4
6. meta-anal:.ti,tw.	6. random:.ti,tw.
7. metanal:.ti,tw.	7. 5 and 6
8. systematic review?.ti,tw.	8. exp meta-analysis/
9. systematic overview?.ti,tw.	9. meta-analysis.ti,tw.
10. quantitative overview?.ti,tw.	10. (meta-anal: or meta anal:).ti,tw.
11. quantitative synthes#s.ti,tw.	11. (quantitative overview: or quantitative synth:).ti,tw.
12. randomized controlled trials/	12. (systematic review: or systematic overview:).ti,tw.
13. randomized controlled trial.pt.	13. exp practice guideline/
14. random allocation/	14. practice guideline.ti,tw.
15. double-blind method/	15. or/8–14
16. single-blind method/	16. 7 or 15
17. random:.ti,tw.	17. exp prostate tumor/
18. controlled clinical trial.pt.	18. exp prostate cancer/
19. clinical trial, phase iii.pt.	19. (prostat: cancer or prostat: carcinoma: or prostat: tumo?*r*: or prostat: malignan:).ti,tw.
20. or/1–19	20. *prostate tumor/dt
21. leuprolide.ti,tw.	21. *prostate cancer/dt
22. lupron.ti,tw.	22. or/17–21
23. goserelin.ti,tw.	23. total androgen blockade.ti,tw.
24. zoladex.ti,tw.	24. maximal androgen blockade.ti,tw.
25. buserelin.ti,tw.	25. androgen ablation.ti,tw.
26. suprefact.ti,tw.	26. flutamide.ti,tw.
27. flutamide.ti,tw.	27. eulexin.ti,tw.
28. eulexin.ti,tw.	28. nilutamide.ti,tw.
29. nilutamide.ti,tw.	29. anandron.ti,tw.
30. anandron.ti,tw.	30. nilandron.ti,tw.
31. nilandron.ti,tw.	31. bicalutamide.ti,tw.
32. bicalutamide.ti,tw.	32. casodex.ti,tw.
33. casodex.ti,tw.	33. cyproterone acetate.ti,tw.
34. cyproterone acetate.ti,tw.	34. androcur.ti,tw.
35. androcur.ti,tw.	35. diethylstilbestrol.ti,tw.
36. diethylstilbestrol.ti,tw.	36. des.ti,tw.
37. des.ti,tw.	37. exp gonadorelin/
38. total androgen blockade.ti,tw.	38. exp androgen antagonists/
39. maximal androgen blockade.ti,tw.	39. exp diethylstilbestrol/
40. combined androgen blockade.ti,tw.	40. or/23–39
41. androgen ablation.ti,tw.	41. exp castration/
42. exp gonadorelin/	42. castration.ti,tw.
43. exp androgen antagonists/	43. orchidectomy.ti,tw.
44. exp diethylstilbestrol/	44. orchiectomy.ti,tw.
45. or/21–44	45. monotherapy.ti,tw.
46. exp castration/	46. leuprolide.ti,tw.
47. castration.ti,tw.	47. lupron.ti,tw.
48. orchidectomy.ti,tw.	48. goserelin.ti,tw.
49. orchiectomy.ti,tw.	49. zoladex.ti,tw.
50. monotherapy.ti,tw.	50. buserelin.ti,tw.
51. or/46–50	51. suprefact.ti,tw.
52. prostatic neoplasms/	52. or/41–51
53. prostat: cancer.ti,tw.	53. 22 and 40 and 52
54. *prostatic neoplasms/dt	54. 53 and 16
55. or/52–54	
56. 45 and 51 and 55	
57. 56 and 20	
